# Exercise as therapy for Parkinson's?

**DOI:** 10.18632/aging.101503

**Published:** 2018-07-20

**Authors:** Erin K. Crowley, Yvonne M. Nolan, Aideen M. Sullivan

**Affiliations:** 1 Department of Anatomy and Neuroscience, University College Cork, Cork, Ireland

**Keywords:** Parkinson's, exercise, α-synuclein, animal model

Although Parkinson's disease (PD) has been traditionally classified as a motor disorder, growing evidence recognises that several non-motor symptoms (NMS) are inherent to the disease, including gastro-intestinal, neuropsychiatric, cognitive and olfactory disturbances. Moreover, some of these symptoms have been shown to be present for up to 15 years prior to the diagnosis of PD [1]. Practically every PD patient suffers from several NMS, to varying extents and with differing profiles, during the course of their disease. These symptoms affect a broad range of physiological systems, have a significant impact upon patients' quality of life and often require multiple pharmacological therapies. Given that PD is an age-related disorder, and that many of the NMS symptoms present at advanced disease stages, the patients with the greatest need of these therapies are elderly. Increasing polypharmacy in an aged population is not desirable due to the associated adverse effects. Thus, there is a clear and growing need for non-pharmacological interventions that can address both the motor and NMS of PD.

Physical exercise has repeatedly been demonstrated to alleviate comorbidities associated with aging, and to contribute to reducing an individual's risk of developing neurodegenerative conditions such as PD or Alzheimer's disease (AD) [2]. The importance of physical exercise for reducing the prevalence of non-communicable diseases has been highlighted by the World Health Organisation, in their 2010 publication, "Global Recommendations on Physical Activity for Health". Moreover, evidence has accumulated to suggest that exercise can ameliorate many of the symptoms of PD, not only the motor dysfunction, but also some of the NMS, such as cognitive impairment and depression [3]. However, in order to decipher the cellular and molecular mechanisms underlying the potential beneficial effects of exercise in PD, it is necessary to employ animal models. The most common models used by the scientific community focus on replicating the motor symptoms of the disease, by applying a chemical lesion in order to cause degeneration of the nigrostriatal dopaminergic pathway, which is responsible for controlling movement. However, such models are not useful for examining the NMS, which typically involve several different neurotransmitter pathways and multiple regions of the brain. Preclinical studies to date utilising exercise as either a neuroprotective or neurorestorative intervention have reported conflicting results in terms of both behavioural and molecular outputs [4]. A recently-developed animal model, involving induction of αsynuclein overexpression in the adult rat brain using adeno-associated viral (AAV) vectors, is widely considered to most consistently reproduce the pathological features and progressive neurodegeneration associated with human PD [5]. However, to date there have been no reports on the use of this model to interrogate potential effects of exercise on PD symptoms.

In our recent study [6], we addressed this by employing the AAV-α-synuclein rat model to test whether exercise could positively impact upon the development of motor and/or NMS of PD. We focused on the cognitive aspect of NMS such as memory tasks, due to the fact that mild cognitive impairment in PD (PD-MCI) has been shown to be present in up to 43% of patients with PD [7].

Adult male Sprague-Dawley rats were given free access to running wheels in cages (voluntary exercise) from one week after administration of AAV-α-synuclein into the substantia nigra. We found that voluntary exercise had no effect on motor function, measured by performance on the Rotarod apparatus, or on dopaminergic neuronal loss in the substantia nigra. However, overexpression of α-synuclein significantly impaired the ability of the animals to perform hippocampal-associated cognitive tasks. This was associated with deficits in hippocampal neurogenesis, a form of neuroplasticity and a key cellular process underlying learning and memory. Importantly, voluntary exercise protected against this cognitive dysfunction, and this protective effect was mediated, at least in part, by alterations in neurogenesis levels.

This is the first study to date that has employed the AAV-α-synuclein model to investigate exercise as a therapeutic intervention, and its strength lies in the fact that this model is widely accepted to be the most similar to the progressive nature of the human condition. Moreover, we have shown at least one mechanism that is involved in exercise-induced neuroprotection. However, it is likely that several distinct mechanisms are at play; further work is needed to define these processes and their relative contributions, in order to explore specific therapeutic targets for PD. It must be appreciated that there are difficulties associated with measuring the effects of exercise in patients, as well as in animal models, that have problems with their motor function. Nevertheless, all of the available evidence suggests a growing rationale for including structured exercise programmes as part of a patient's therapeutic regimen. Exercise should be incorporated into a holistic personalised treatment programme that is tailored to each patient's specific needs and, importantly, their motor abilities.
